# Rules of Aseptic and Antiseptic Surgery

**Published:** 1888-03

**Authors:** 


**Rules of Aseptic and Antiseptic Surgery—A Practical Treatise for the Use of Students and the General Practitioner.** By ARPAD G. GERSTER, M. D., Professor of Surgery at the New York Polyclinic; Visiting Surgeon to Mt. Sinai Hospital and the German Hospital, New York. Illustrated with 248 engravings and three chromo-lithographic plates. New York: D. Appleton & Co. 1888.

This is as beautiful a specimen of the book-makers' art as we have seen. By the photo-typographic process, 248 photographs have been reproduced to illustrate the text; and for the proper presentation of these, the publishers have printed the entire work upon highly-calendered paper, so thick as to resemble card-board. The typography and mechanical work is as near perfection as can be attained. We congratulate Dr. Gerster that his work has been issued by the Appletons—for the author's work commands the highest commendation, and deserves a perfect presentation. This work does not pretend to be a systematic treatise on surgery, but its object is a systematic yet practical presentation of the Listerian principle that has revolutionized surgery within the last fifteen years. Nelaton was in the habit of saying that the man who discovered a method of preventing purulent infection would deserve to have erected in his honor a statue of gold. To-day, it is a definitely-settled scientific fact that suppuration cannot be caused by chemical irritants. Where pus is, these micro-organisms have been at work; or, in other words, without the action of living bacteria, there can be no suppuration. From this it follows, as a necessary sequence, that, with our present knowledge of the principle of asepticism, the infection and suppuration of a fresh wound are due to faults of omission or commission on the part of the surgeon. We frequently hear the statement that the principle of Listerism is simply cleanliness; but ordinary cleanliness is not asepsis. What chemical purity is to the chemist, and sterilization to the bacteriologist, so is surgical cleanliness or antisepsis to the surgeon, and this kind of cleanliness can only be reached through scientific antisepsis. Listerism is, however, to many a chaos of seemingly contradictory and incomprehensible details; its rules varied by the whims of this or that teacher.

To these, and indeed to all surgeons, this book is valuable, because it gives the practical directions of an adept, and because it is largely a record of personal experience. The beauty and abundance of the illustrations—which are photographs taken during operation—add greatly to the practical value of the work. In a word, it is a book which every physician who does any surgical work ought to have.

